# Proposal for universality in the viscosity of metallic liquids

**DOI:** 10.1038/srep13837

**Published:** 2015-09-09

**Authors:** M. E. Blodgett, T. Egami, Z. Nussinov, K. F. Kelton

**Affiliations:** 1 Department of Physics and Institute of Materials Science and Engineering, Washington University, St. Louis, MO 63130 USA; 2 Joint Institute for Neutron Sciences, University of Tennessee, Knoxville TN 37996 and Oak Ridge National Laboratory, Oak Ridge TN 37830 USA

## Abstract

The range of magnitude of the liquid viscosity, *η*, as a function of temperature is one of the most impressive of any physical property, changing by approximately 17 orders of magnitude from its extrapolated value at infinite temperature (*η*_o_) to that at the glass transition temperature, *T*_g_. We present experimental measurements of containerlessly processed metallic liquids that suggest that log(*η/η*_o_) as a function of *T*_A_/*T* is a potentially universal scaled curve. In stark contrast to previous approaches, the scaling requires only two fitting parameters, which are on average predictable. The temperature *T*_A_ corresponds to the onset of cooperative motion and is strongly correlated with *T*_g_, suggesting that the processes underlying the glass transition first appear in the high temperature liquid.

The nature of the dynamical processes in liquids and how liquids transform to glasses are major outstanding questions in condensed matter science. The shear viscosity is a particularly temperature-sensitive property for glass-forming liquids, changing by about 17 orders of magnitude upon cooling from high temperatures to the glass transition temperature, *T*_*g*_. The way in which the viscosity, or related relaxation times, change with temperature scaled to *T*_g_ is the basis for the widely-used *fragility* classification scheme introduced by Angell^1^. For very *strong* liquids the viscosity shows an Arrhenius behavior, with a well-defined activation energy over a wide temperature range that extends from above the melting temperature down to *T*_g_. The viscosities of fragile liquids are characterized by activation energies that are small at high temperature and increase rapidly upon approaching *T*_g_. The strongest glass-formers are network oxides, while molecular liquids such as o-terphenyl, decalin and isoquinoline, are among the most fragile. Upon close examination, some non-Arrhenius behavior is observed near the glass transition, even in strong liquids, but this becomes more dramatic as the fragility of the liquid increases. Thermodynamic^2^ and direct structural signatures^3^ of fragility support a connection between structure and dynamics in liquids, which has been long assumed. The concept of fragility appears to provide a coherent scheme for classifying all liquids and linking to glass formability in some cases. However, in spite of the significant variation in fragility among various liquid metals, the viscosity experimental data for those presented here are remarkably more universal. They suggest the existence of a high-temperature universal scaling temperature, *T*_A_, which has been predicted from molecular dynamics (MD) simulations of metallic liquids^4^ and theoretical studies of non-metallic glass-forming liquids^5^,^6^,^7^. Scaling the temperature, *T,* by *T*_A_ (a temperature that corresponds to the onset of dynamical cooperativity in the liquid), and scaling the viscosity by *η*_o_ (the extrapolated high temperature limit on the viscosity of the liquid phase) yields an apparently universal curve that fits the viscosities of all liquid metals studied, from above the melting temperature to the glass transition temperature.

## Results

### Measurements of the Liquid Viscosity and a Universal Curve

**Figure 1:**
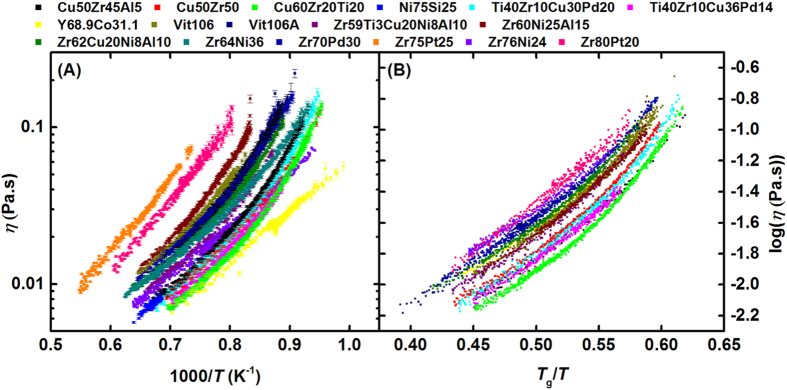
(**A**) Measured viscosity, *η*, as a function of temperature for metallic liquids. Error bars are one s.d. (**B**) An Angell plot of the log(*η*) as a function of *T*_g_/*T*.

The viscosities of a variety of metallic liquids were measured at high temperatures in a high-vacuum containerless environment, using electrostatic levitation (see Supplementary Information). These are shown as a function of inverse temperature in Fig. 1A. The liquids studied include liquids that easily form metallic glasses, such as Zr_57_Cu_15.4_Ni_12.6_Al_10_Nb_5_ (Vit 106) and Zr_58.5_Cu_15.6_Ni_12.8_Al_10.3_Nb_2.8_ (Vit 106A), and more marginal glass-forming fragile liquids, such as Ti-Zr-Cu-Pd and Cu-Zr, where faster cooling rates are required for glass formation. Also included are liquids for which glass formation has not been observed, such as Ni-Si. Figure 1B shows an Angell plot for these data, presenting log(*η*) as a function of *T*_g_/*T*. Scaling the temperature by *T*_g_ reduces the scatter of the data from Fig. 1A, but significant variations among the data remain.

**Figure 2:**
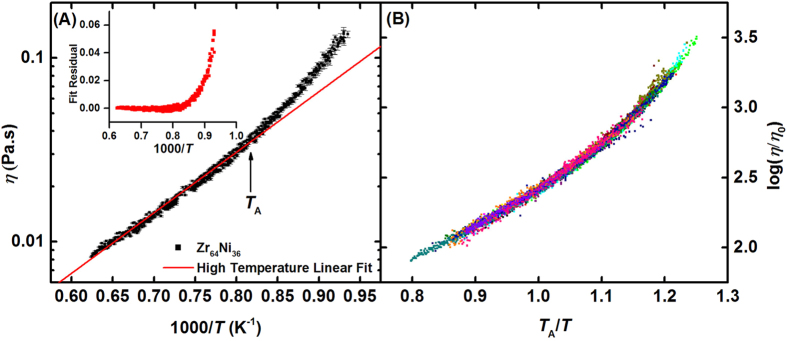
(**A**) Typical example of the behavior of log(*η*) at high temperature, showing a departure from Arrhenius behavior on cooling below *T*_A_. Error bars are one s.d. (**B**) Scaled universal curve for all measured viscosity data.

As illustrated in Fig. 2A for a Zr_64_Ni_36_ liquid, the viscosities of all of the data in Fig. 1 have Arrhenius temperature dependences at a sufficiently high temperature. This agrees with the results from previous studies^5^,^8^,^9^ and recent MD simulations for several different types of metallic liquids^4^. The temperature at which the measured viscosity departs from Arrhenius behavior is labeled in Fig. 2A as *T*_A_. While the departure is gradual and difficult to determine directly, it becomes clearer in the residuals from a linear fit (insert to figure). The physical meaning of *T*_A_ is intriguing; from MD simulations^4^ it corresponds to the temperature at which flow first becomes cooperative.

The MD simulations revealed a universal curve for the ratio of the Maxwell relaxation time for viscosity and the time required to change the local coordination number in a cluster by one unit, by scaling the temperature with the temperature corresponding to the onset of dynamical cooperativity (defined there also as *T*_A_). Those results suggested that the measured viscosity data could be scaled by *T*_A_. More recent simulation results reveal concurrent cooperative structural changes beginning at *T*_A_^10^. As is verified in Fig. 2B, by adopting two material-dependent parameters for scaling, *T*_A_ and *η*_o_, all of the data can be collapsed into a universal curve that describes the temperature dependent viscosity of the liquids studied. To construct this curve, the scaling temperature, *T*_A,_ was determined for each liquid as illustrated in Fig. 2A, defined as the temperature below which the viscosity became non-Arrhenius. The value of *η*_o_ for each liquid was then adjusted to collapse the data along the vertical axis. For all liquids studied, on average *η*_o_ ≈ *nh*^11^,^12^, where *n* is the particle density and *h* is Planck’s constant. Exact values and comparisons to *nh* can be found in Supplementary Table 2.

### Functional Form of the Universal Curve

**Figure 3:**
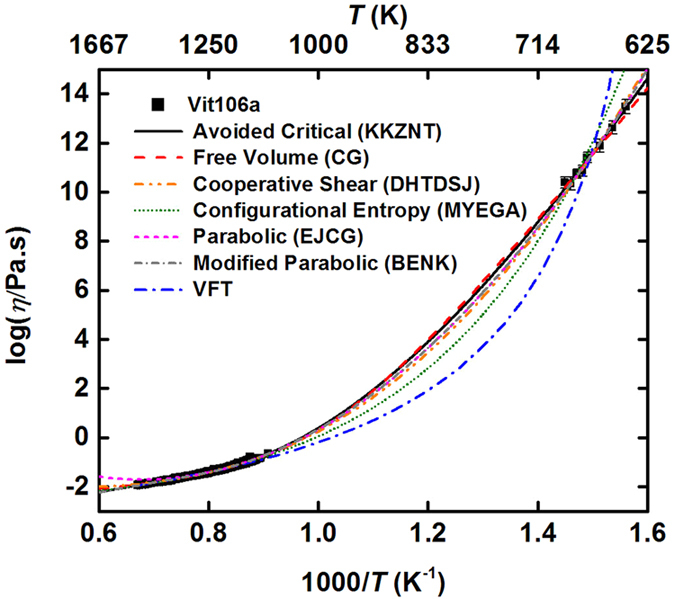
Comparison of the fits to the Vit 106a data for the expressions listed in Table 1. The fit parameters can be found in Supplementary Table 1. Error bars are one s.d.

While it is shown that our measured data lie on a universal curve when properly scaled, the temperature range is small. An immediate consequence of the observed data collapse is that, at intermediate temperatures, *η* = *η*_o_*F*(*T*/*T*_A_). Here, *F*(*z*) is assumed to be a universal function common to all of the metallic liquids studied (i.e., one exhibiting *no adjustable parameters*). To examine whether the scaling holds over a larger temperature range, measured viscosity data near *T*_g_ for the strong metallic glass-forming liquid Vit 106a^13^ were combined with the high temperature data reported here for the same liquid. However, since the scaling in Fig. 2B is empirical, extending it to lower temperatures requires knowledge of the functional form of the universal curve. Many earlier proposed expressions for the viscosity exist, but it has not been previously possible to extensively test them in metallic liquids over the wide temperature range that is possible here, extending from above the melting temperature to the glass transition temperature. The combined Vit 106a data, then, constitute a benchmark for testing these expressions and for identifying the one (if any) that describes the universal behavior. The fits to the Vit 106a data for some of the better-known expressions are shown in Fig. 3. These are (i) the commonly used Vogel-Fulcher-Tammann (VFT) equation^14^, (ii) the recently proposed Mauro-Yue-Elliston-Gupta-Allan (MYEGA) equation^15^, (iii) a relation derived within the Cohen-Grest free volume model (CG)^16^, (iv) the avoided critical point theory (KKZNT)^5^,^6^,^7^, (v) a cooperative shear model (DHTDSJ)^17^, and (vi) a parabolic kinetically constrained model (EJCG)^18^,^19^. Since the EJCG expression is only valid up to its onset temperature *T*_o_, leaving higher temperature behavior undefined, a new variant (BENK) is introduced here, for which the constant Arrhenius type barrier is augmented to give to an expression that emulates that of the avoided critical point theory when *T* is replaced by 1/*T*. Its crossover temperature 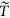 is similar to T^*^ in the KKZNT expression. These equations are defined in Table 1 and the corresponding optimal parameters are listed in Supplementary Table 1. Other approaches^20^,^21^,^22^ that are associated with scaling temperatures typically near the mode-coupling temperature, below our experimentally accessible temperature region, could not be examined.

All of these expressions fit the high-temperature viscosity data reasonably well, although most do not fit the entire temperature range. In particular, the most-commonly used VFT, and, to a lesser degree the MYEGA, expressions are both in poor agreement with the slope of the data near *T*_g_ (672K). Further, in the high temperature limit the MYEGA form approaches an Arrhenius temperature dependence far more weakly than predicted by the MD simulations^4^ and our experimental data. As a consequence, the fit values for *η*_o_ are much larger than from fits to the other models and from the scaled data shown in Fig. 2B. The CG, DHTDSJ, BENK, and KKZNT models all fit the data over the entire temperature range. The DHTDSJ expression and to a lesser extent the CG form are, however, not consistent with a cross-over to a high temperature Arrhenius type behavior.

**Figure 4:**
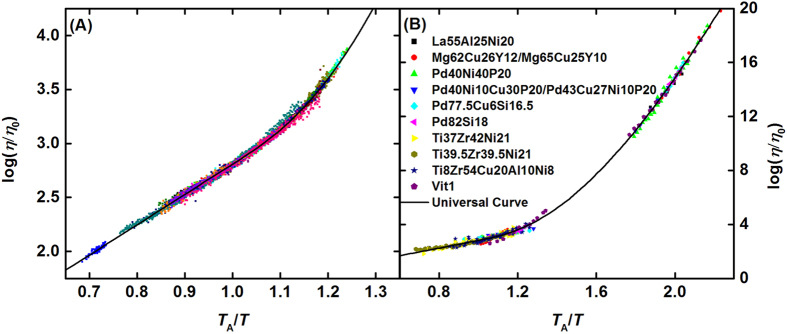
(**A**) Collapse of viscosity data from Fig. 1 onto a universal curve assuming the avoided critical point form (KKZNT, black curve). Supplementary Figure 2 shows the residuals of the fit. (**B**) Data collapse of measurements reported in this work and for literature data for additional metallic glass-forming liquids. See Supplementary Table 2 for references to the data for the additional liquids broken down by composition.

We now discuss a particular approximate expression to the universal function *F*(*z*) appearing in our data collapse. In earlier studies, the avoided critical point expression (KKZNT) was shown to fit the viscosity data for many non-metallic liquids^5^,^6^,^7^, albeit with five fitting parameters, compared to the three or four parameters for the other models investigated here. The expression may be written as *η* = *η*_0_exp(*E/T*) with a free energy barrier (expressed here in Kelvin) *E* = *E*_∞_ + *T*_A_ (*bT*_*r*_)^*z*^Θ(*T*_A_ − *T*), where Θ(*x*) is the Heaviside function and the “reduced temperature” is *T*_*r*_  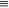 (*T*_A_ − *T*)/*T*_A_. Earlier considerations suggested that some of those parameters are fixed. Bolstered by theory, empirical tests^5^,^6^,^7^ yielded an exponent z ≈ 8/3 ± 1/3. The KKZNT expression includes an “avoided critical point temperature” T^*^, which like *T*_A_ in the MD results and the scaling temperature *T*_A_ introduced here, corresponds to the onset temperature for dynamical cooperativity. Based on theoretical considerations^7^, T^*^/*T*_*l*_ = 1.08 for idealized liquids, where *T*_*l*_ is the liquidus, or melting, temperature—a tendency that is on average, is obeyed, but with significant spread. The universal curve further constrains the KKZNT expression, such that *T*_A_ and *η*_o_ are the only remaining free parameters, with the values of the other parameters (now constants) determined by the fit to Vit 106a: *E*_∞_ = 6.466*T*_A_, *b* = 4.536, *z* = 2.889. As shown in Fig. 4A, with *T*_A_ and *η*_o_ as the only free parameters this expression gives an excellent fit to all of the high temperature viscosity data shown in Fig. 1. The values for *T*_A_ and *η*_o_ obtained from the fits are listed in Supplementary Table 2. The residuals of the fit are also plotted in Supplementary Figure 2. These *T*_A_ values are roughly 10% higher than those determined manually, with some scatter of a few percent. That the human eye would pick a lower temperature where the curvature is more apparent is unsurprising; the scatter is due to the difficulty in picking a temperature by eye in some alloys (e.g., Zr-Pt). These fit values are predictable from those obtained from the empirical scaling procedure used to find the universal curve for the high temperature data (Fig. 2B). Additionally, as will be demonstrated shortly, *T*_A_ ≈ 2.02*T*_g_ and (with less precision) *η*_o_ ~ *nh*. Thus, the KKZNT fits to the universal collapse are nearly parameter free. As shown in Fig. 4B, this same constrained expression also fits data obtained by other investigators^17^,^23^,^24^,^25^,^26^,^27^,^28^,^29^,^30^,^31^,^32^,^33^ over a wide range of metallic glass families. Excluding a section for which measurements cannot be currently made, the universal curve fits well over 16 orders of magnitude in the viscosity. The reason for the small deviation in the high temperature data for Vit 1^17^ is likely due to a reported fragility transition in that liquid^34^; no similar deviation was observed in our measured data for other liquids. Such good agreement for a range of different metallic liquids provides a striking demonstration of the validity of the KKZNT expression as an approximate functional form for the universal curve. Nevertheless, the possibility of other reasonable approximate forms is not ruled out. For example, the BENK expression (Table 1) also gives good agreement. The precise functional form for *η* = *η*_o_*F*(*T*/*T*_A_) is a matter for future theoretical studies. However, the KKZNT gives sufficiently good agreement with the experimental data over a very wide temperature range that it can be used here to examine the nature of the experimental scaling parameters.

**Figure 5:**
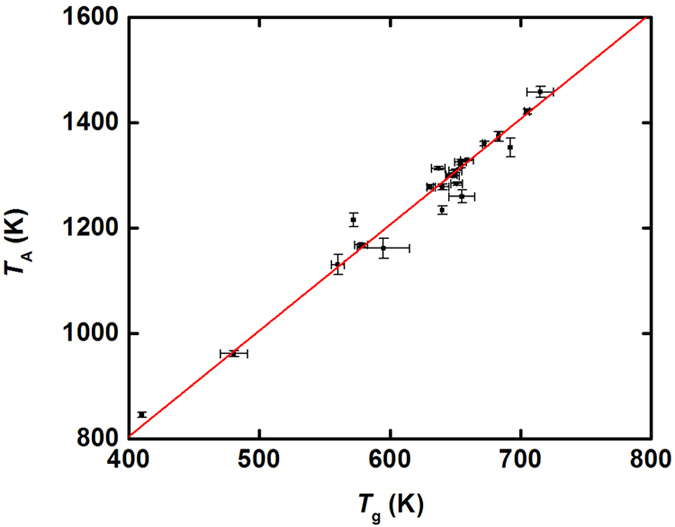
Comparison between the experimentally measured glass transition temperature, *T*_*g*_, and *T*_*A*_ from the fits to the high temperature viscosity data. The solid line is a fit to the data, giving *T*_*A*_ = (2.02 ± 0.015)*T*_*g*_. Error bars are one s.d.

Figure 5 shows the correlation between the values of *T*_A_ and *T*_g_ for the glass-forming liquids. The *T*_g_ values were obtained by us and by others from differential scanning calorimetry measurements, using a range of heating rates from 10 to 40 K per minute. The fit line shows that *T*_A_/*T*_g_ = 2.02 ± 0.015. This correlation is remarkable, and suggests a deep connection between the onset of cooperative dynamics in the liquid and the dynamical slowing down at the glass transition temperature. It is also the reason that *T*_g_ remains a useful scaling temperature.

This correlation also suggests novel approaches for the search for good glass forming liquids, at least for metallic glasses. For example, assuming the Turnbull criterion of good glass formability when (*T*_g_/*T*_l_) is large^35^, makes it possible to assess trends in glass formability from liquid data alone, without actually forming a glass and measuring *T*_*g*_. A study of the change in *T*_A_ values with the chemical composition of the liquid would show whether such a survey is practically useful.

### Further Discussion of the Scaling Parameters

As discussed, the scaling parameters *η*_o_ and *T*_A_ can be obtained from fits to the KKZNT theory. However, it is important to underscore that they can also be determined empirically by collapsing all of the experimental data onto a universal curve, making them fundamentally theory independent. Several interesting points emerge from an examination of the values obtained for the parameters.

The values for the extrapolated high-temperature viscosity, *η*_o_, suggest that there may exist a universal high-temperature limit of the viscosity. For the liquid metals studied here, this is *on average* equal to *nh* (<*η*_o_> = *nh*)^12^, where *h* is Plank’s constant and *n* is the particle number per unit volume and the brackets <> denote an average over all of the liquids studied. Such typical values for *η*_o_ have been predicted^11^,^12^ and may have an even deeper significance that extends beyond that of liquid metals. Fundamental lower limits on the viscosity are discussed widely in various contexts, with recent interest^36^ driven by predictions from string theory and holographic dualities, which were compared with measurements at the Relativistic Heavy Ion Collider^37^.

The strong correlation between *T*_A_ and *T*_g_ (*T*_A_ ~2.02 *T*_g_) for all of the metallic-glass-forming liquids examined supports a long-held belief from other complementary approaches that glass formation might be a consequence of a high temperature transition crossover. Theories of this crossover include an avoided critical point^5^,^6^,^7^, a random first order transition^38^,^39^, and mode coupling theories^40^, among others (e.g.^41^). In the avoided critical point theory *T*_A_ corresponds to the transition temperature of the supercooled liquid in an idealized template – a transition that is avoided by frustration. There have been previous experimental hints of non-trivial dynamics associated with a viable dynamical crossover temperature, *T*_cross_ (above *T*_g_). These include (a) the appearance (at *T* < *T*_cross_) of short time, or *β*, relaxation processes accompanying the primary, or *α*, relaxation rates that are the focus of this work^42^, (b) the broadening of relaxation times about these two principal processes (typically this broadening is manifest in response functions that have a stretched exponential behavior)^20^,^43^, (c) nonuniform dynamics in different spatial regions (dynamical heterogeneities)^44^, (d) violation of the Stokes-Einstein relation^45^, and (e) decoupling of translational and rotational diffusivity^46^, and (f) phonon localization^4^. These phenomena appear and are strongly indicative of transformations that have an onset above *T*_g_, yet at temperatures that are lower than 2 *T*_g_ in most studied non-metallic liquid systems. It is also important to note that *T*_A_ is much above predicted dynamic crossover temperatures^41^ and the mode-coupling temperature^47^.

### Emerging Questions

The results presented here raise several questions. For example, what is the origin of the observed universal behavior and the connection between *T*_g_ and *T*_A_? It has long been known that many supercooled metallic liquids veer towards locally preferred low energy icosahedral structures^5^,^6^,^7^,^48^,^49^,^50^, a tendency that other liquids generally do not share. This general propensity towards locally preferred structures lies at the origin of the avoided critical point model^5^,^6^,^7^. MD simulations show that on decreasing the temperature below *T*_A_ many metallic glass forming liquids progressively develop more pronounced icosahedral order, with a length scale of connected icosahedral cluster networks that monotonically increases until they percolate throughout the entire system near *T*_g_ (see, e.g., Figure 7 of Ref. 10). This is in agreement with predications from avoided critical point theory^5^,^6^,^7^, and could be the source of the connection between *T*_g_ and *T*_A_ that is experimentally observed here. Additionally, over the ensemble of metallic liquids that were examined, *T*_A_/*T*_l_ ≈ 1.075 ± 0.188 (where *T*_l_ is the liquidus temperature), consistent with estimates suggested by the avoided critical theory^7^. However, it should be emphasized that local order need not be icosahedral for theories of an avoided critical point nature to be valid. The local structures of Pd-Si, for example, are likely not strongly icosahedral, while some liquids that have more definitive icosahedral order and still fit the universal curve (e.g., Zr-Pt) deviate more from the predictions of the scaling parameters *η*_o_ and *T*_A_ than do others.

A second question is what these results imply about liquid fragility. Metallic liquids show a range of fragility, but occupy the central part of an Angell plot^1^. The stronger metallic liquids (e.g. Vit106) are more fragile than strong liquids like SiO2. If *η*_o_ were truly independent of temperature for liquid metals, as suggested from the data presented, and if *T*_A_/*T*_g_ were truly constant, then the fragility index, defined as *m* 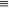 (∂log_10_*η*/∂(*T*_g_/*T*))_*T*_ = _*T*g_, would be the same for all metallic liquids. This is extremely unlikely, however. Evidence for fragility exists not only in the dynamical properties (where typically an assumed VFT form for *η* is invoked), but also in thermodynamic properties^51^,^52^ and rate of structural ordering^3^,^53^. Our studies show that fragility is embedded in the nature of the deviation of the *T*_A_/*T*_g_ ratio found for different metallic liquids as well as possible remnant small deviations of the viscosity data from a universal collapse. It is useful to see how the fragility index *m* varies with *T*_A_/*T*_g_ for all of the liquids studied. For metallic liquids fragility is frequently determined from calorimetric measurements^54^ in addition to or when viscosity data are unobtainable. However, while this gives reliable values of *m* for good glass-formers, the values for marginal glass forming liquids are scarce and often unreliable. This makes it impossible at present to examine the correlation for all of the liquids studied. Instead, Supplementary Fig. 1 compares the ratio *T*_A_/*T*_g_ with values of *m* reported^13^,^55^,^56^,^57^ for the good glass-forming liquids studied here with. The *m* values range from about 32 to 75; for comparison the *m* values for SiO_2_ and o-terphenyl are 20 and 81, respectively. While there are substantial disparities in the values of *m* that different groups have reported for any single metallic liquid, the average reported fragility index values clearly increase with *T*_A_/*T*_g_, in agreement with a structural origin of fragility (see Ref. 53). However, as seen from the steepness of the slope in Figure S1, the large variations in the values of *m* evaluated just above *T*_g_ are not similarly reflected in the *T*_A_/*T*_g_ ratio. The origin of this enigmatic behavior is not understood.

## Summary and Conclusions

In summary, the measured data suggest a new universality in the dynamical behavior of liquid metals when the temperature is scaled by *T*_A_, which corresponds to the onset of dynamical cooperativity, and when the extrapolated infinite temperature viscosities (*η*_o_) are properly accounted for. That the glass transition temperature, *T*_g_, is strongly correlated with *T*_A_ suggests that the cooperative processes that eventually lead to the glass transition are already present in the high temperature liquid. The rapid cooling rates needed for molecular dynamics (MD) studies often raise questions of the validity of comparisons between computed results and experimental data for temperatures near *T*_g_. The results presented here, however, show that it is possible to more realistically probe processes associated with the glass transition by comparing computed results with experimental data obtained at high-temperature, where experimental and MD relaxation times are comparable. It remains to be seen whether the results and conclusions presented here extend to liquids other than metallic ones, but our data for the metallic liquids clearly motivate the need for further investigation.

## Additional Information

**How to cite this article**: Blodgett, M. E. *et al.* Proposal for universality in the viscosity of metallic liquids. *Sci. Rep.* **5**, 13837; doi: 10.1038/srep13837 (2015).

## Supplementary Material

Supplementary Information

## Figures and Tables

**Table 1:** Tested Fitting Functions for Viscosity.

Vogel-Fulcher-Tammann (VFT)	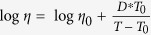
Configurational Entropy (MYEGA)	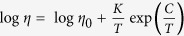
Free Volume (CG)	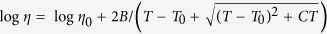
Avoided Critical (KKZNT)	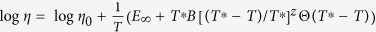
Cooperative Shear (DHTDSJ)	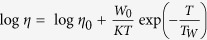
Parabolic (EJCG)	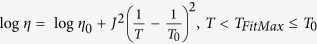
Modified Parabolic (BENK)	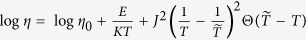

Θ(*x*) is the Heaviside function (i.e., Θ(*x* > 0) = 1 and Θ(*x* < 0) = 0).
