# Supplementary material for: Proposal for universality in the viscosity of metallic liquids
Source: Sci Rep. 2015 Sep 9;5:13837. doi: 10.1038/srep13837 (PMC4563367; doi:10.1038/srep13837)
Supplement: Supplementary Information [file srep13837-s1.docx]

**Proposal for universality in the viscosity of metallic liquids**

**Supplementary Information**

M. E. Blodgett, T. Egami, Z. Nussinov and K. F. Kelton

**Materials & Methods**

The samples were prepared from master-alloys that were made by arc-melting on a water cooled hearth in a high-purity argon (99.998%) argon environment. Elements of high purity - 99.9% (Y & Co) to 99.9999% (Cu) - were used to prepare the alloys. When possible, source material was selected for minimum oxygen content (e.g. <10 ppm Zirconium), as this dramatically affects the amount of super-cooling attainable. A Ti-Zr getter was also melted before arc-melting the elements to further reduce the residual oxygen in the atmosphere. The approximately one gram master ingots were melted three times to ensure a homogenous composition; ingots with mass loss greater than 0.05% were discarded. The master ingots were then broken apart and re-melted into samples for the Electrostatic Levitation Studies (ESL); these were in a mass range 40-90 mg.

Samples were then levitated and melted in the high-vacuum containerless environment of the Washington University Beamline ElectroStatic Levitation Facility (WU-BESL). The absence of a container and the high-vacuum environment (~10^-7^ Torr) minimized heterogeneous nucleation, allowing data to be collected from both equilibrium and supercooled liquids. More details of the WU-BESL can be found elsewhere^1^.

The viscosity was measured as a function of temperature using an oscillating drop method^2^. The voltage on the vertical electrode was modulated at a frequency that near the *l* = 2 spherical harmonic mode resonant frequency (typically 120–140 Hz) of the liquid to induce surface vibrations. A high-speed camera (1560 frames per second) was used to capture the shadow of the oscillating sample. After the oscillation was stable, the perturbative voltage was removed and the time-dependent amplitude of the decaying surface harmonic oscillations was measured. The viscosity was determined from the decay time for the oscillation, τ,

*η* = *ρR_0_*/(*l* -1)(2*l* +1)τ

where *ρ* is the density and *R_0_* is the unperturbed radius of the sample. The small magnitude of the viscosity over the measurement range and the low strain rates ensure that shear thinning does not influence the measurements.

**Supplementary Table 1**

Values of Parameter from Fits to Vit106A, as Shown in Figure 2 and Table 1 of the Main Text.

| **Fitting Equation** | **log_10_(η_0_/Pa.s))** | **Other Parameter Values** |
| --- | --- | --- |
| **Vogel-Fulcher-Tammann (VFT)** | -3.48 | D^*^=5.60, T_0_=575 |
| **Configurational Entropy (MYEGA)** | -2.80 | K=237, C=2.49x10^3^ |
| **Free Volume (CG)** | -2.67 | B=873, C=58.6, T_0_=954 |
| **Avoided Critical (KKZNT)** | -4.50 | E_∞_=3819, T^*^=1360, B=34.3, z=2.889 |
| **Cooperative Shear (DHTDSJ)** | -2.11 | W_0_=1.36 x10^5^, T_W_=247 |
| **Parabolic (EJCG)** | -1.72 | J^2^=1.97 x10^7^, T_0_=1476 |
| **Modified Parabolic (BENK)** | -4.64 | E=4.01x10^3^, J^2^=1.96x10^7^, =1285 |

**Supplementary Table 2**

The Scaling Parameters and *T*_A_, their Relation to the Predicted High Temperature Viscosity Limit (*nh*) and the Glass Transition Temperature (*T*_g_), and High Temperature Activation Energy (E_∞_).

| **Composition** | *Log_10_(nh)* | *Log_10_(η_0_)* | ***±1σ*** | ***T*_A_** | ***±1σ*** | ***T*_g_** | ***E_∞_*** |
| --- | --- | --- | --- | --- | --- | --- | --- |
|  | *(Pa.s)* | *(Pa.s)* |  | *K* | *K* | *K* | *eV* |
| Cu_50_Zr_45_Al_5_ | -4.44 | -4.60 | 0.01 | 1308 | 3.4 | 650 | 0.7290 |
| Cu_50_Zr_50_ | -4.45 | -4.60 | 0.01 | 1284 | 2.7 | 651 ^3*^ | 0.7152 |
| Cu_60_Zr_20_Ti_20_ | -4.39 | -4.73 | 0.01 | 1301 | 2.5 | 647 | 0.7247 |
| Ni_75_Si_25_ | -4.29 | -4.15 | 0.22 | 1072 | 120 | - | 0.5971 |
| Ti_40_Zr_10_Cu_30_Pd_20_ | -4.41 | -4.61 | 0.01 | 1299 | 3.7 | 648 | 0.7236 |
| Ti_40_Zr_10_Cu_36_Pd_14_ | -4.40 | -4.64 | 0.02 | 1278 | 5.6 | 640 | 0.7118 |
| Vit106^†^ **^4^** **^*^** | -4.48 | -4.45 | 0.02 | 1373 | 9.0 | 683 ^4 *^ | 0.7651 |
| Vit106A^†^ **^4 *^** | -4.48 | -4.50 | 0.01 | 1360 | 4.0 | 672 ^4 *^ | 0.7577 |
| Y_68.9_Co_31.1_ | -4.57 | -4.40 | 0.05 | 1130 | 19 | 560 | 0.6296 |
| Zr_59_Ti_3_Cu_20_Ni_8_Al_10_ | -4.50 | -4.52 | 0.02 | 1320 | 6.0 | 652 | 0.7357 |
| Zr_60_Ni_25_Al_15_ | -4.50 | -4.50 | 0.01 | 1421 | 5.0 | 698 | 0.7916 |
| Zr_62_Cu_20_Ni_8_Al_10_ | -4.50 | -4.45 | 0.01 | 1325 | 4.7 | 654 | 0.7380 |
| Zr_64_Ni_36_ | -4.49 | -4.25 | 0.03 | 1223 | 17 | - | 0.6817 |
| Zr_70_Pd_30_ | -4.53 | -4.39 | 0.01 | 1329 | 1.0 | 659 | 0.7402 |
| Zr_75_Pt_25_ | -4.50 | -4.40 | 0.01 | 1550 | 0.8 | - | 0.8637 |
| Zr_76_Ni_24_ | -4.52 | -4.25 | 0.04 | 1161 | 19 | 595 | 0.6470 |
| Zr_80_Pt_20_ | -4.41 | -4.36 | 0.02 | 1458 | 10 | 715 ^5 *^ | 0.8257 |
| **Literature Data** |  |  |  |  |  |  |  |
| La_55_Al_25_Ni_20_ ^6,7 *^ | -4.64 | -5.02 | 0.18 | 966.4 | 6.0 | 481 ^8 *^ | 0.5355 |
| Mg_62_Cu_26_Y_12_ ^9^ ^*^/ Mg_65_Cu_25_Y_10_ ^10 *^ | -4.47 | -4.35 | 0.12 | 854.0 | 3.8 | 410 ^10 *^ | 0.4732 |
| Pd_40_Ni_40_P_20_ ^6,7,11 *^ | -4.32 | -4.82 | 0.08 | 1168 | 2.8 | 578 ^6 *^ | 0.6506 |
| Pd_40_Ni_10_Cu_30_P_20_ ^12 *^/ Pd_43_Ni_10_Cu_27_P_20_ ^13 *^ | -4.31 | -5.23 | 0.09 | 1215 | 8.2 | 572 ^13 *^ | 0.6775 |
| Pd_82_Si_18_ ^14,15 *^ | -4.41 | -4.87 | 0.04 | 1277 | 4.2 | 631 | 0.7117 |
| Pd_77.5_Cu_6_Si_16.5_ ^7 *^ | -4.41 | -4.87 | 0.07 | 1313 | 3.1 | 637 | 0.7316 |
| Ti_37_Zr_42_Ni_21_ ^16 *^ | -4.49 | -4.33 | 0.04 | 1177 | 12 | - | 0.6555 |
| Ti_39.5_Zr_39.5_Ni_21_ ^17 *^ | -4.48 | -4.18 | 0.03 | 1144 | 13 | - | 0.6372 |
| Ti_8_Zr_54_Cu_20_Al_10_Ni_8_ ^18 *^ | -4.49 | -4.34 | 0.05 | 1259 | 12 | 655 | 0.7017 |
| Vit1^†^ ^7 *^ | -4.47 | -3.30 | 0.07 | 1242 | 3.0 | 613 ^7 *^ | 0.6919 |

^*^References to viscosity and calorimetry data obtained by other investigators.

^†^ Vit106 [Zr_57_Cu_15.4_Ni_12.6_Al_10_Nb_5_], Vit106a [Zr_58.5_Cu_15.6_Ni_12.8_Al_10.3_Nb_2.8_], Vit1 [Zr_41.2_Ti_13.8_Cu_12.5_Ni_10_Be_22.5_]


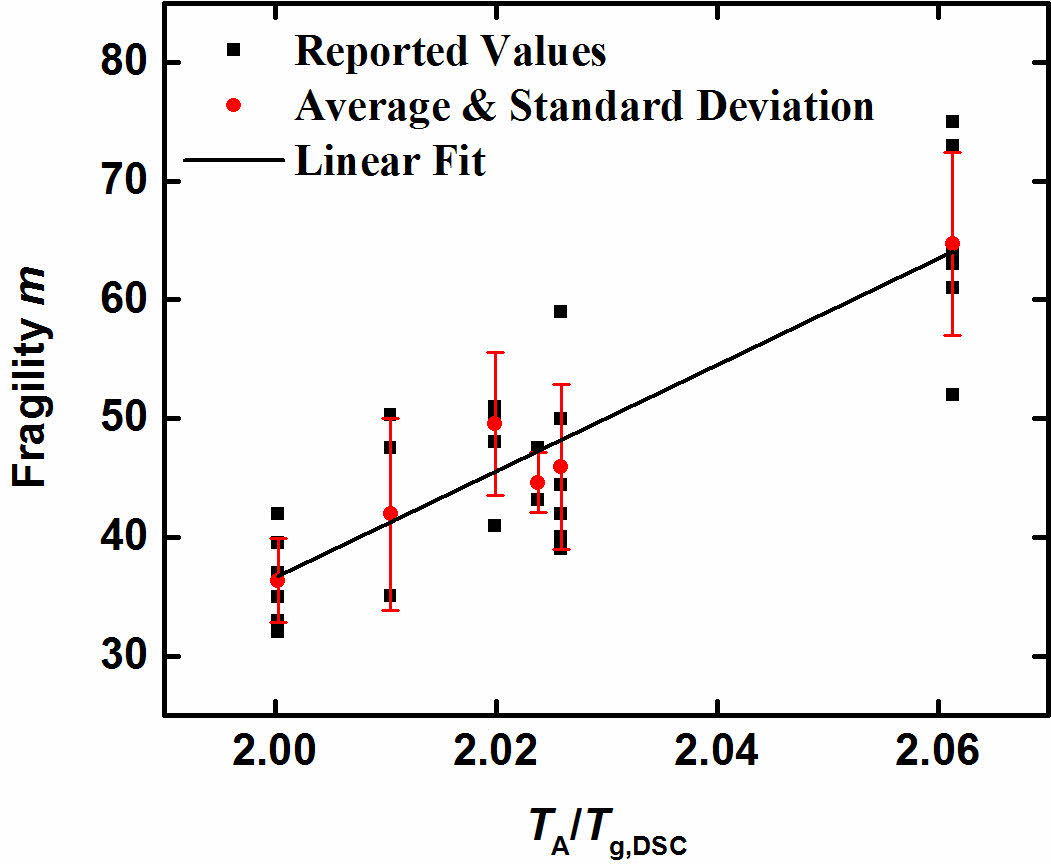


**Supplementary Figure 1 –** Published fragility (*m*) data (◼) versus *T*_A_/*T*_g_. The filled red circles (⚫) represent the average values; the error bars reflect the standard deviation. The large scatter in the reported *m* values for these bulk metallic glasses, as well as the lack of data for marginal glass-formers, reflects the difficulty in measuring *m*. From left to right the compositions are La_55_Al_25_Ni_20_^19-21^, Vit106^4^, Pd_40_Ni_40_P_20_^19-21^, Vit106a^4^, Vit1^19-21^, Pd_77.5_Cu_6_Si_16.5_^19-21^.


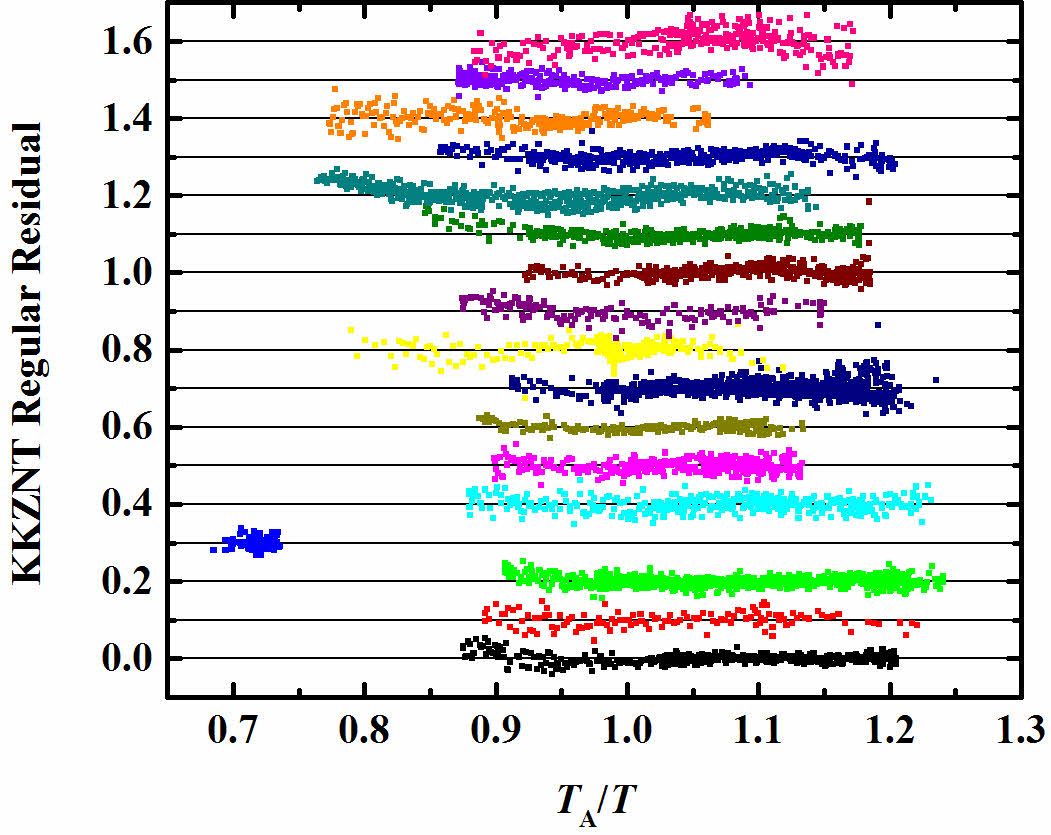


**Supplementary Figure 2** – Residual scatter of the data in Figure 4a after subtracting the KKZNT fit. An offset of 0.1 was added between data sets for clarity. In two cases (Zr_80_Pt_20_, Zr_64_Ni_36_) the data appear to deviate slightly from the KKZNT fit. This may be experimental error. It may also indicate a small deviation from the correlation of *T*_A_ with the high temperature activation energy (ratio equal to 2.88 K/ev), which was found in the fit to Vit106a data and assumed to be constant for all of these metallic liquids in order to minimize the number of free parameters. Allowing this ratio to be fit for each metallic liquid removes the few cases for deviation in the residual plot, but gives an average value that differs only slightly from that for Vit 106a (2.76 K/ev). Also, the correlation coefficients for the fits show only marginal improvement (from 0.9829 to 0.9887 for Zr_80_Pt_20_, for example).

**Supplementary References**

1 Mauro, N. A. & Kelton, K. F. A highly modular beamline electrostatic levitation facility, optimized for in situ high-energy x-ray scattering studies of equilibrium and supercooled liquids. *Rev Sci Instrum* **82**, 35114-35116, doi:10.1063/1.3554437 (2011).

2 Rhim, W. K., Ohsaka, K., Paradis, P.-F. o. & Spjut, R. E. Noncontact technique for measuring surface tension and viscosity of molten materials using high temperature electrostatic levitation. *Rev Sci Instrum* **70**, 2796-2801, doi:10.1063/1.1149797 (1999).

3 Yu, P., Bai, H. Y. & Wang, W. H. Superior glass-forming ability of CuZr alloys from minor additions. *J Mater Res* **21**, 1674-1679, doi:10.1557/Jmr.2006.0212 (2006).

4 Evenson, Z., Raedersdorf, S., Gallino, I. & Busch, R. Equilibrium viscosity of Zr–Cu–Ni–Al–Nb bulk metallic glasses. *Scr Mater* **63**, 573-576, doi:10.1016/j.scriptamat.2010.06.008 (2010).

5 Saida, J. *et al.* Nano quasicrystal formation and local atomic structure in Zr–Pd and Zr–Pt binary metallic glasses. *Z Kristallogr* **223**, 726-730, doi:10.1524/zkri.2008.1041 (2008).

6 Kawamura, Y., Nakamura, T., Kato, H., Mano, H. & Inoue, A. Newtonian and non-Newtonian viscosity of supercooled liquid in metallic glasses. *Mat Sci Eng A-Struct* **304**, 674-678, doi:10.1016/S0921-5093(00)01562-8 (2001).

7 Demetriou, M. D. *et al.* Cooperative shear model for the rheology of glass-forming metallic liquids. *Phys Rev Lett* **97**, 065502, doi:10.1103/PhysRevLett.97.065502 (2006).

8 Okumura, H., Chen, H. S., Inoue, A. & Masumoto, T. Sub-Tg mechanical relaxation of a La_55_Al_25_Ni_20_ amorphous alloy. *J Non-Cryst Solids* **130**, 304-310, doi:10.1016/0022-3093(91)90367-F (1991).

9 Wu, S.-S., Chin, T.-S., Su, K.-C. & Shyr, F.-S. Undercooled Liquid Viscosity and Glass Formation of a Mg_62_Cu_26_Y_12_Alloy. *Japanese Journal of Applied Physics* **35**, 175-178, doi:10.1143/JJAP.35.175 (1996).

10 Busch, R., Liu, W. & Johnson, W. L. Thermodynamics and kinetics of the Mg_65_Cu_25_Y_10_ bulk metallic glass forming liquid. *J App Phys* **83**, 4134, doi:10.1063/1.367167 (1998).

11 Tsang, K. H., Lee, S. K. & Kui, H. W. Viscosity of molten Pd_40_Ni_40_P_20_. *J App Phys* **70**, 4837-4841, doi:10.1063/1.349050 (1991).

12 Haumesser, P.-H., Bancillon, J., Daniel, M., Perez, M. & Garandet, J.-P. High-temperature contactless viscosity measurements by the gas–film levitation technique: Application to oxide and metallic glasses. *Rev Sci Instrum* **73**, 3275-3285, doi:10.1063/1.1499756 (2002).

13 Lu, I. R., Görler, G. P., Fecht, H. J. & Willnecker, R. Investigation of specific volume of glass-forming Pd–Cu–Ni–P alloy in the liquid, vitreous and crystalline state. *J Non-Cryst Solids* **312-314**, 547-551, doi:10.1016/S0022-3093(02)01767-2 (2002).

14 Tsang, K. H. & Kui, H. W. Viscosity of molten Pd_82_Si_18_ and the scaling of viscosities of glass forming systems. *J App Phys* **72**, 93-96, doi:10.1063/1.352101 (1992).

15 Stojanova, L., Russew, K. & Illekova, E. Study of the Structural Relaxation of Pd82si18 Metallic-Glass by Thermal-Expansion and Viscous-Flow Measurements. *Mat Sci Eng A-Struct* **133**, 529-531, doi:10.1016/0921-5093(91)90126-8 (1991).

16 Hyers, R. *et al.* Surface tension and viscosity of quasicrystal-forming Ti–Zr–Ni alloys. *Int J Thermophys* **25**, 1155-1162 (2004).

17 Bradshaw, R. C. *et al.* Nonlinearities in the undercooled properties of Ti_39.5_Zr_39.5_Ni_21_. *Phil Mag* **86**, 341-347, doi:10.1080/14786430500253968 (2006).

18 Bradshaw, R. C. *et al.* Containerless Measurements of Thermophysical Properties of Zr_54_Ti_8_Cu_20_Al_10_Ni_8_. *Ann N Y Acad Sci* **1077**, 63-74, doi:10.1196/annals.1362.058 (2006).

19 Perera, D. N. Compilation of the fragility parameters for several glass-forming metallic alloys. *J Phys: Condens Matter* **11**, 3807-3812, doi:10.1088/0953-8984/11/19/303 (1999).

20 Johnson, W. L., Demetriou, M. D., Harmon, J. S., Lind, M. L. & Samwer, K. Rheology and ultrasonic properties of metallic glass-forming liquids: A potential energy landscape perspective. *MRS bulletin* **32**, 644-650 (2007).

21 Takeuchi, A., Kato, H. & Inoue, A. Vogel-Fulcher-Tammann plot for viscosity scaled with temperature interval between actual and ideal glass transitions for metallic glasses in liquid and supercooled liquid states. *Intermetallics* **18**, 406-411, doi:10.1016/j.intermet.2009.08.015 (2010).
